# Long Noncoding RNA MALAT1 Promotes Laryngocarcinoma Development by Targeting miR-708-5p/BRD4 Axis to Regulate YAP1-Mediated Epithelial-Mesenchymal Transition

**DOI:** 10.1155/2022/8093949

**Published:** 2022-05-12

**Authors:** Xiaoqin Wu, Yenong Tan, Xuxia Tang

**Affiliations:** ^1^Department of Otolaryngology, Hainan Provincial Hospital of Traditional Chinese Medicine, Haikou, Hainan, China; ^2^Department of Otolaryngology, The First Affiliated Hospital of Zhejiang Chinese Medical University, Hangzhou, Zhejiang, China

## Abstract

**Objective:**

The objective of this study was to investigate whether long noncoding RNA Metastasis-Associated Lung Adenocarcinoma Transcript 1 (MALAT1) contributes to laryngocarcinoma development via regulating the Yes-associated protein 1- (YAP1-) mediated epithelial-mesenchymal transition (EMT) and the underlying mechanism.

**Methods:**

The effects of MALAT1 suppression and BET inhibitor JQ1 on the malignant phenotypes and cancer stem cell- (CSC-) like properties of laryngocarcinoma cells as well as the expression of bromodomain-containing protein 4 (BRD4), YAP1, and EMT markers were investigated. Moreover, the relationships between MALAT1 and miR-708-5p as well as between miR-708-5p and BRD4 were explored. Furthermore, whether MALAT1 regulated the malignant phenotypes of laryngocarcinoma cells via sponging miR-708-5p to target BRD4 was revealed by both *in vitro* and *in vivo* experiments.

**Results:**

MALAT1 suppression inhibited the malignant phenotypes of laryngocarcinoma cells, such as decreased proliferation, promoted apoptosis, suppressed migration, and inhibited the CSC properties. Suppression of MALAT1 increased miR-708-5p expression and decreased the expression of BRD4 and YAP1 and inhibited EMT. Moreover, there were target relationships between MALAT1 and miR-708-5p as well as between miR-708-5p and BRD4. miR-708-5p overexpression and MALAT1 suppression had synergistic inhibitory effects on the malignant phenotypes of laryngocarcinoma cells and the expression of BRD4, YAP1, and EMT. Furthermore, *in vivo* experiments confirmed that MALAT1/miR-708-5p regulated tumorigenicity by regulating BRD4 and YAP1-mediated EMT.

**Conclusions:**

Our results indicate that suppression of MALAT1 may inhibit laryngocarcinoma development by sponging miR-708-5p/BRD4 to regulate YAP1-mediated EMT. Targeting MALAT1/miR-708-5p/BRD4 axis may provide a promising therapeutic strategy for laryngocarcinoma.

## 1. Introduction

Laryngocarcinoma is a type of common malignant tumor of the head and neck worldwide [1]. The incidence and mortality of laryngocarcinoma is high, causing about 83,000 deaths per year worldwide [2]. Due to lack of effective early diagnosis biomarkers, most laryngocarcinoma are diagnosed at an advanced stage [3], and the 5-year survival rate of patient with laryngocarcinoma is less than 50% [4]. Therefore, uncovering the crucial molecular mechanism underlying laryngocarcinoma carcinogenesis is highly desired for exploring promising diagnostic biomarkers and effective treatment strategies.

Long noncoding RNAs (lncRNAs) are a class of noncoding RNAs longer than 200 bp [4, 5]. LncRNAs can play a significant role in many cellular functions and are widely involved in the initiation and metastasis of human cancers [5–7]. In laryngocarcinoma, several lncRNAs, such as PCAT19 [8], NEAT1 [9], and LOXL1-AS1 [10], function as key players in malignant progression. Moreover, an increasing number of studies indicate that aberrantly expressed lncRNAs have been exploited as potential biomarkers for cancer diagnosis and prognosis [11, 12]. Metastasis-Associated Lung Adenocarcinoma Transcript 1 (MALAT1), a 6.5-knt lncRNA, has been found to be involved in multiple steps in the development of tumors and exhibits diagnostic and prognostic significance in diverse cancers, such as prostate cancer, glioma, hepatocellular carcinoma, breast cancer, lung cancer, and multiple myeloma [13, 14]. However, the crucial roles and regulatory mechanism of MALAT1 in laryngocarcinoma remain unclear.

Yes-associated protein 1 (YAP1) plays a significant role in malignancies, which can promote malignant phenotypes and drug resistance of cancer cells and facilitate expansion of cancer stem cells (CSCs) [15]. Overexpression of YAP1 can induce epithelial-mesenchymal transition (EMT) and enhance cell migration and invasion in colorectal cancer cells [16]. Moreover, bromodomain-containing protein 4 (BRD4), one of the major bromodomain and extraterminal motif (BET) family members, is revealed as a critical regulator for YAP1 transcription and expression [17]. However, the function of BRD4 and YAP1 in laryngocarcinoma development has not been reported. Notably, accumulating evidence has reported that lncRNAs contribute to disease development via functioning as competitive endogenous RNAs (ceRNAs) to regulate gene expression by sponging miRNAs [18, 19]. Whether MALAT1 contributes to laryngocarcinoma development via sponging miRNA to regulate BRD4 expression is largely unknown.

In the present study, we first investigated the effect of MALAT1 suppression and BET inhibitor JQ1 on the malignant phenotypes and CSC-like properties of laryngocarcinoma cells as well as the expression of BRD4, YAP1, and EMT markers. Moreover, we detected whether MALAT1 could act as a ceRNA to regulate the development of laryngocarcinoma both *in vitro* and *in vivo*. Our findings will improve our understanding of the mechanism of laryngocarcinoma and provide a new insight for diagnosis and treatment of this disease.

## 2. Materials and Methods

### 2.1. Cell Culture

The laryngocarcinoma cell line Hep-2 was obtained from the American Type Culture Collection (ATCC, Manassas, VA, USA). Hep-2 cells were cultured in Dulbecco's modified Eagle's medium (DMEM, Hyclone, Logan, Utah) supplemented with 10% fetal bovine serum (FBS, Gibco, Sydney, Australia) at 37°C in the atmosphere of 5% CO_2_. JQ1, solubilized in DMSO, was diluted into media at 1 mM and incubated with cells for 2 h at 37°C.

### 2.2. Cell Transfection

Short hairpin RNA-targeting MALAT1 (sh-MALAT1, GTAACTGGCATGTGAGCAA) and negative control shRNA (sh-NC) were designed and chemically synthesized by the Hanbio Co. Ltd. (Shanghai, China). The lentiviral vector expressing sh-MALAT1 and sh-NC was constructed and then transfected into Hep-2 cells. Moreover, the miR-708-5p mimics and negative control (miR-NC) were transfected into Hep-2 cells using Lipofectamine 2000 (Invitrogen, Carlsbad, CA, USA).

### 2.3. CCK-8 Assay

Hep-2 cells in different groups were collected and then plated into a 96-well plate. At indicated time points (0, 24, 48, and 72 h), 10 *μ*L CCK-8 solution (Beyotime, Jiangsu, China) was added into each well to incubate cells for 2 h at 37°C. The absorbance value at 450 nm wavelength was measured using a microplate reader (Bio-Rad, Hercules, USA).

### 2.4. Flow Cytometry for Apoptosis Detection

For detection of cell apoptosis, Hep-2 cells in different groups were harvested after 0.25% trypsin digestion. Cell apoptosis was then detected using the annexin V-FITC/PI apoptosis detection kit (Beyotime, Jiangsu, China) following the manufacturers' instruction. Briefly, cells were stained at room temperature and in the dark with 5 *μ*L annexin V-FITC for 15 min and 10 *μ*L propidium iodide (PI) for 10 min. The percentage of cell apoptosis was detected with a FACSCalibur flow cytometer and analyzed by CELL Quest software (Becton Dickinson, Mountain View, CA).

### 2.5. Transwell Migration Assay

Hep-2 cells in different groups were harvested after 0.25% trypsin digestion and then resuspended with serum-free media with the cell density adjusted to 1 × 106/mL. Cell suspension (200 *μ*L) was added into the upper chamber (8 mm pore size; Millipore, Billerica, MA, USA) of Transwell inserts. The lower chamber was filled with 600 *μ*L complete medium containing 20% FBS. After incubation for 24 h at 37°C, the chambers were fixed with formaldehyde for 30 min and air-dried properly. Then, the chambers were stained with 0.1% crystal violet for 20 min. The unmigrated cells in the upper chamber were gently wiped with cotton swab and rinsing three times with PBS. The migrated cells in the lower chamber were observed in five random fields under a 400x microscope (IX71, Olympus, Tokyo, Japan) and counted.

### 2.6. Real-Time Quantitative Reverse Transcription PCR (qRT-PCR)

Total RNA was isolated from Hep-2 cells in different groups using Trizol (Invitrogen). Reverse transcription to cDNA was carried out using PrimeScriptTM RT Master Mix Kit (Takara). Real-time qRT-PCR was then performed using a qPCR Kit (SYBR Premix Ex Taq) (TaKaRa). The PCR conditions included an initial degeneration step at 95°C for 3 min, followed by 40 times cycle of denaturation at 95°C for 12 s and extension at 62°C for 40 s. Relative gene expression was calculated using the 2^-*ΔΔ*Ct^ method.

### 2.7. Western Blot Assay

Hep-2 cells in different groups were lysed in RIPA buffer (TaKaRa, Japan) containing PMSF (Sigma-Aldrich, Shanghai, China) for 15 min at 4°C. After centrifugation, the supernatants were collected as the protein extracts. The concentration of protein extracts was detected using BCA method (Thermo Scientific, Waltham, USA). After separated on 10% SDS-polyacrylamide gels, the protein blots were transferred onto polyvinylidene fluoride (PVDF) membranes (Millipore, Billerica, MA, USA). The membranes were blocked with 5% skimmed milk and then incubated with primary antibodies to BRD4, YAP1, E-cadherin, N-cadherin, and GAPDH (1 : 1000 dilution; Abcam, Cambridge, UK) overnight at 4°C and HRP-labelled secondary antibodies (Multi sciences) for 60 min at room temperature in the dark. After rinsing three times, the blots were visualized using ECL method (Thermo), and the gray value of protein blots was analyzed using Image J software.

### 2.8. Tumorsphere Formation Assay

To generate tumorspheres, Hep-2 cells in different groups were digested with trypsin, inoculated in a 6-well ultralow adhesion culture plate with the density of 500 cells/well, and then cultured in the serum free medium containing DMEM/F12 (Gibco), 2% B-27, 0.4% bovine serum albumin, 20 ng/mL basal fibroblast growth factor (bFGF), 20 ng/mL epidermal growth factor (EGF), and 5 *μ*g/mL insulin at 37°C in the atmosphere of 5% CO_2_. After 11 days of culture, the number of generated tumorspheres was counted under a microscope.

### 2.9. Analysis of Side Population (SP) Cell Content

Hep-2 cells in different groups were collected and prepared into single-cell suspension. The fluorescent dye Hoechst33342 (5 *μ*g/mL) was added and incubated the cells for 90 min. After rinsing with PBS, the dead cells were labeled with PI. The SP cells that were defined as negative or weak positive Hoechst33342 fluorescence were sorted using a flow cytometer (FACS Aria II; BD Biosciences).

### 2.10. Dual-Luciferase Reporter Assay

To construct the reporter vectors MALAT1-wild-type (MALAT1-WT, agaugcagagaaaacAGCUCCUu) and MALAT1-mutated-type (MALAT1-MUT, agaugcagagaaaacUCGAGGAa) as well as BRD4-wild-type (BRD4-WT, gaCAGCAGCAGCGAUAGCUCCUc) and BRD4-mutated-type (BRD4-MUT, gaGUCGAGCUGCCAUUCGAGGAa), the fragments of MALAT1 or BRD4 containing the predicted miR-708-5p binding site and flanking sequence were, respectively, inserted into the pmirGlO Dual-luciferase miRNA Target Expression Vector (Promega, Madison, WI, USA). The constructed reporter vectors, along with miR-708-5p mimic or mimic control, were then transfected into Hep-2 cells. After 48 h of transfection, the luciferase activity was the detected by the Dual-Luciferase Reporter Assay System (Promega). Renilla luciferase activity was used as an internal control.

### 2.11. In Vivo Experiments

All procedures were performed according to the protocols approved by the Institutional Animal Ethics Care and Use Committee. The 4-week-old BALB/c mice were purchased from Shanghai Ling Chang Biological Technology Co., Ltd. and were housed in sterilized cages. The BALB/c mice were randomly divided into 5 groups, including model, sh-NC, sh-MALAT1, sh-MALAT1+miR-NC, and sh-MALAT1+miR-708-5p agomir groups. To observe the tumourigenicity, 1 × 10^6^ Hep-2 cells transfected with sh-MALAT1, sh-NC, miR-708-5p agomir, and miR-NC were subcutaneously injected into the right flank of mice in each group. Tumor sizes were measured every three days, and tumor volumes were calculated using the following formula: *V*(mm^3^) = (width^2^ × length) × 1/2. At the end of the experiment, the tumor tissue was weighed and frozen for immunohistochemistry (IHC) staining, qRT-PCR, and western blot assays.

### 2.12. IHC Staining

Tumor tissues in different groups were fixed, dehydrated, and paraffin-embedded. Paraffin-embedded sections were then prepared, followed by deparaffining, hydration, and antigen retrieval. The sections were incubated overnight with Ki67 (1 : 600 dilution, Abcam) antibody at 4°C. Subsequently, the sections were incubated with secondary antibody (1 : 1000 dilution, Abcam) for 1 h. After dehydration and mounting, the sections were observed under the microscope.

### 2.13. Statistical Analysis

Statistical analyses were accomplished by the GraphPad Prism (version 7.0; GraphPad Software, La Jolla, California). Data are expressed as the mean ± standard error (SD). The differences between two groups were analyzed using one-way ANOVA. *P* < 0.05 was indicated as statistically significant.

## 3. Results

### 3.1. MALAT1 Suppression or JQ1 Treatment Decreased Proliferation, Promoted Apoptosis, Suppressed Migration, and Inhibited the CSC-like Properties of Laryngocarcinoma Cells

**Figure 1:**
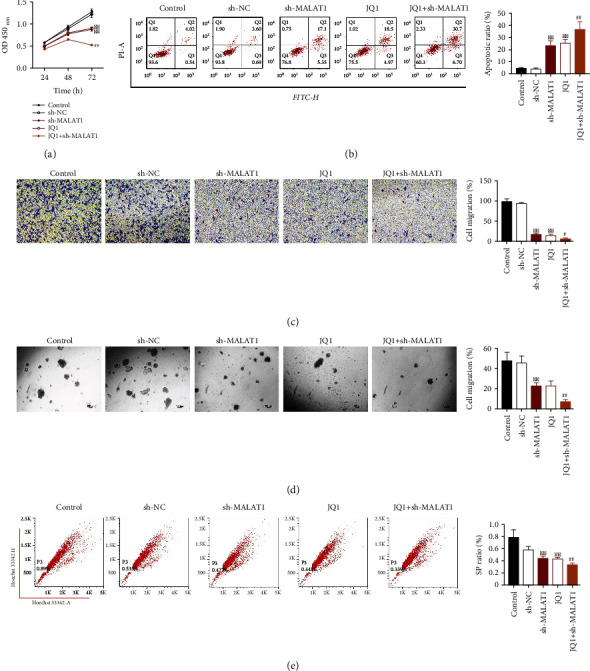
MALAT1 suppression or JQ1 treatment decreased proliferation (a), promoted apoptosis (b), suppressed migration (c), and inhibited the CSC-like properties (tumorsphere formation (d) and side population (SP) cell content (e) of laryngocarcinoma cells. ^∗∗^*P* < 0.01 compared with the control group. ^#^*P* < 0.05 and ^##^*P* < 0.01 compared with the JQ1 group.

CCK8 assay was first performed to detect Hep-2 cell viability of different groups (Figure 1(a)). Compared to the control group, cell viability was significantly inhibited in the sh-MALAT1, JQ1, or JQ1+sh-MALAT1 groups (*P* < 0.01), suggesting that MALAT1 suppression or JQ1 treatment inhibited laryngocarcinoma cell proliferation. Moreover, compared with the JQ1 group, cell viability was further dramatically decreased after combined treatment of MALAT1 suppression and JQ1 (*P* < 0.01).

Cell apoptosis of different groups was detected by flow cytometry (Figure 1(b)). As results, the percentage of Hep-2 cell apoptosis in the sh-MALAT1, JQ1, or JQ1+sh-MALAT1 groups was significantly increased relative to that in the control group (*P* < 0.01). Compared with the JQ1 group, cell apoptosis was further increased after combined treatment of MALAT1 suppression and JQ1 (*P* < 0.01). These data indicated that MALAT1 suppression and/or JQ1 treatment could significantly promote the apoptosis of laryngocarcinoma cells.

Cell migration of different groups was evaluated by Transwell assay. As displayed in Figure 1(c), the number of migrated cells in the sh-MALAT1, JQ1, or JQ1+sh-MALAT1 groups was remarkably decreased in comparison with that in the control group (*P* < 0.01), and there was no obvious change in cell status between control and sh-NC groups (*P* > 0.05). These data confirmed that that MALAT1 suppression and/or JQ1 treatment could significantly repress the migration of laryngocarcinoma cells.

In addition, the effects of MALAT1 suppression or JQ1 treatment on the CSC-like properties of laryngocarcinoma cells were investigated. The results of tumorsphere formation assay showed that the number of generated tumorspheres of laryngocarcinoma cells in the sh-MALAT1, JQ1, or JQ1+sh-MALAT1 groups was obviously decreased compared with the control group (Figure 1(d)). Moreover, the results of flow cytometry revealed that MALAT1 suppression and/or JQ1 treatment resulted in the significant decreases in SP cell content (*P* < 0.01), and the inhibitory effect on SP cell content was the most significant in the JQ1+sh-MALAT1 group (Figure 1(e)). These data demonstrated that MALAT1 suppression and/or JQ1 treatment inhibited the CSC-like properties of laryngocarcinoma cells.

### 3.2. Suppression of MALAT1 Increased miR-708-5p Expression and Decreased the Expression of BRD4, YAP1, and EMT

**Figure 2:**
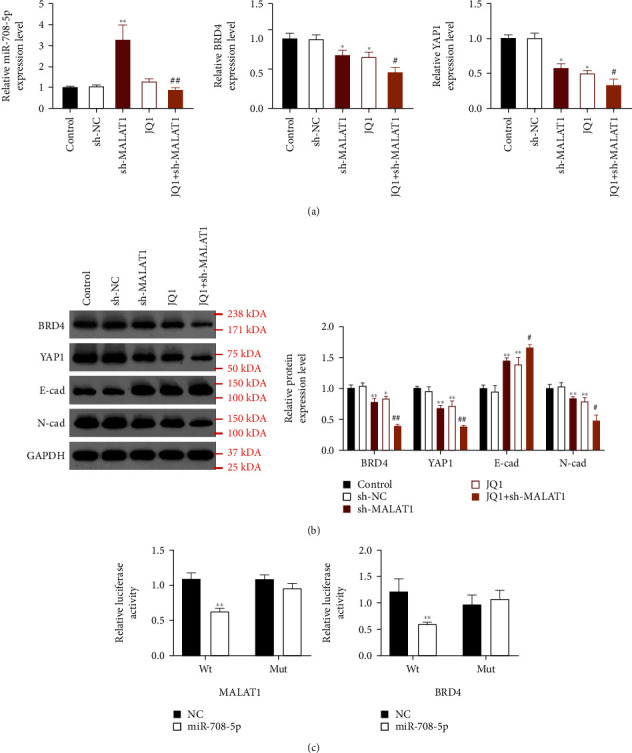
qRT-PCR (a) and western blot (b) assay revealed that suppression of MALAT1 increased miR-708-5p expression and decreased the expression of BRD4, YAP1, and EMT. ^∗^*P* < 0.05 and ^∗∗^*P* < 0.01 compared with the control group. ^#^*P* < 0.05 and ^##^*P* < 0.01 compared with the JQ1 group. (c) Dual-luciferase reporter assays revealed that there were target regulatory relationship between MALAT1 and miR-708-5p as well as between miR-708-5p and BRD4. ^∗∗^*P* < 0.01 compared with the NC group.

To explore the possible mechanism of MALAT1 in laryngocarcinoma cells, we first determined the expression of miR-708-5p, BRD4, and YAP1 by qRT-PCR. As results, we found that compared with the control group, sh-MALAT1 treatment significantly increased miR-708-5p experssion levels (*P* < 0.01). The BRD4 and YAP1 mRNA expression was markedly decreased in the sh-MALAT1, JQ1, or JQ1+sh-MALAT1 groups compared to the control group (*P* < 0.05, Figure 2(a)). Consistently, the results of western blot also showed that the protein expression levels of BRD4 and YAP1 were remarkably downregulated in the sh-MALAT1, JQ1, or JQ1+sh-MALAT1 groups in comparison with the control group (*P* < 0.05, Figure 2(b)). In addition, the expression levels of EMT markers (E-cadherin and N-cadherin) were investigated and the results showed that N-cadherin protein expression was significantly decreased in the sh-MALAT1, JQ1, or JQ1+sh-MALAT1 groups, while E-catenin protein expression was dramatically increased (*P* < 0.05, Figure 2(b)). These data suggest that the suppression of MALAT1 might affect the expression of miR-708-5p, BRD4, YAP1, and EMT markers. To further reveal whether there were regulatory relationships between MALAT1 and miR-708-5p as well as between miR-708-5p and BRD4, dual-luciferase reporter assays were performed. The results showed that the luciferase activity of MALAT1-Wt and BRD4-Wt groups was significantly decreased after miR-708-5p administer (*P* < 0.01, Figure 2(c)), indicating the target regulatory relationship between MALAT1 and miR-708-5p as well as between miR-708-5p and BRD4.

### 3.3. The Malignant Phenotypes of Laryngocarcinoma Cells Were Inhibited after Overexpression of miR-708-5p, which Were Further Decreased after miR-708-5p Overexpression and MALAT1 Suppression at the Same Time

**Figure 3:**
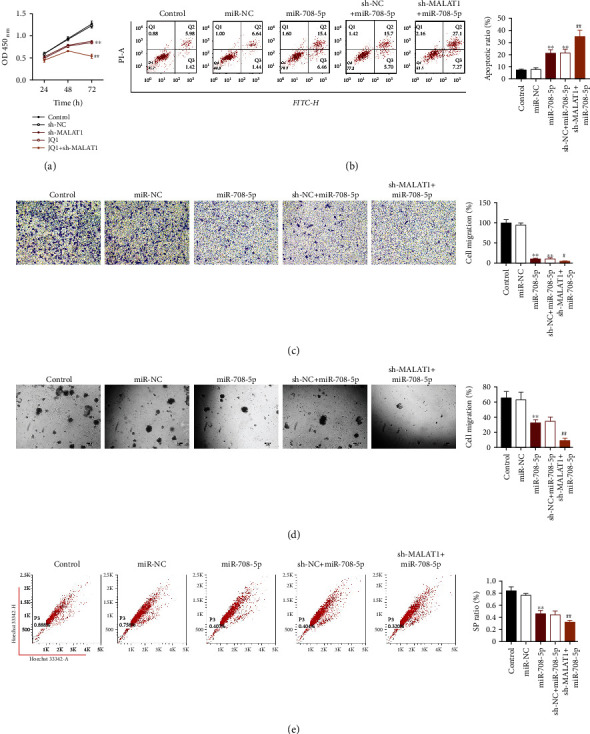
Overexpression of miR-708-5p significantly inhibited proliferation (a), promoted apoptosis (b), suppressed migration (c), and inhibited the CSC-like properties (d) and (e) of laryngocarcinoma cells, which were further decreased after miR-708-5p overexpression and MALAT1 suppression at the same time. ^∗∗^*P* < 0.01 compared with the control group. ^#^*P* < 0.05 and ^##^*P* < 0.01 compared with the sh-NC+miR-708-5p ago group.

We further investigated the effects of miR-708-5p overexpression and combined treatment of miR-708-5p overexpression and MALAT1 suppression on the proliferation, apoptosis, migration, and CSC-like properties of laryngocarcinoma cells. The results showed that overexpression of miR-708-5p significantly inhibited proliferation (Figure 3(a)), promoted apoptosis (Figure 3(b)), suppressed migration (Figure 3(c)), and inhibited the CSC-like properties (Figures 3(d) and 3(e)) of laryngocarcinoma cells, which were further decreased after miR-708-5p overexpression and MALAT1 suppression at the same time (*P* < 0.01).

### 3.4. miR-708-5p Overexpression and MALAT1 Suppression Had Synergistic Inhibitory Effects on the Expression of BRD4, YAP1, and EMT

**Figure 4:**
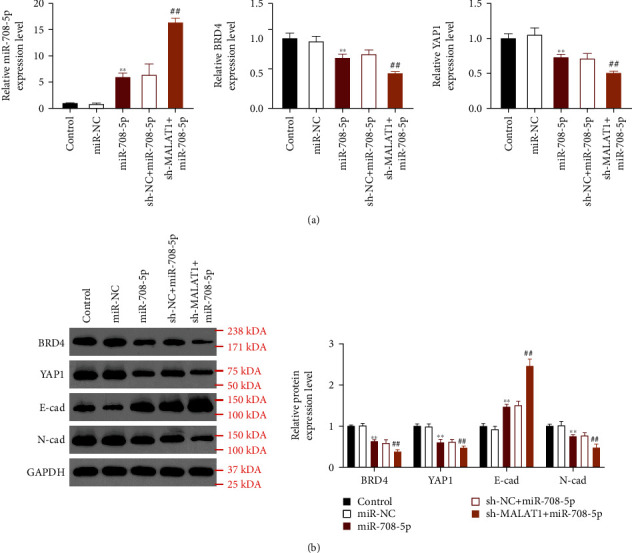
qRT-PCR (a) and western blot (b) assay revealed that miR-708-5p overexpression and MALAT1 suppression had synergistic inhibitory effects on the expression of BRD4, YAP1, and EMT. ^∗∗^*P* < 0.01 compared with the control group. ^#^*P* < 0.05 and ^##^*P* < 0.01 compared with the sh-NC+miR-708-5p ago group.

The results of qRT-PCR showed that compared with the NC group, overexpression of miR-708-5p in miR-708-5p ago group significantly increased the expression of miR-708-5p and decreased the mRNA expression of BRD4 and YAP1; moreover, compared to the sh-NC+miR-708-5p ago group, the expression changes of miR-708-5p, BRD4, and YAP1 were further enhanced after combination of miR-708-5p overexpression and MALAT1 suppression in the sh-MALAT1+miR-708-5p ago group (all *P* < 0.01, Figure 4(a)). In addition, the results of western blot showed that the protein expression of BRD4, YAP1, and N-cadherin was significantly downregulated, while the E-cadherin protein expression was dramatically up- and downregulated after overexpression of miR-708-5p, which were further aggravated after combination of miR-708-5p overexpression and MALAT1 suppression (all *P* < 0.01, Figure 4(b)).

### 3.5. MALAT1/miR-708-5p Regulated Tumorigenicity In Vivo by Regulating BRD4 and YAP1-Mediated EMT

**Figure 5:**
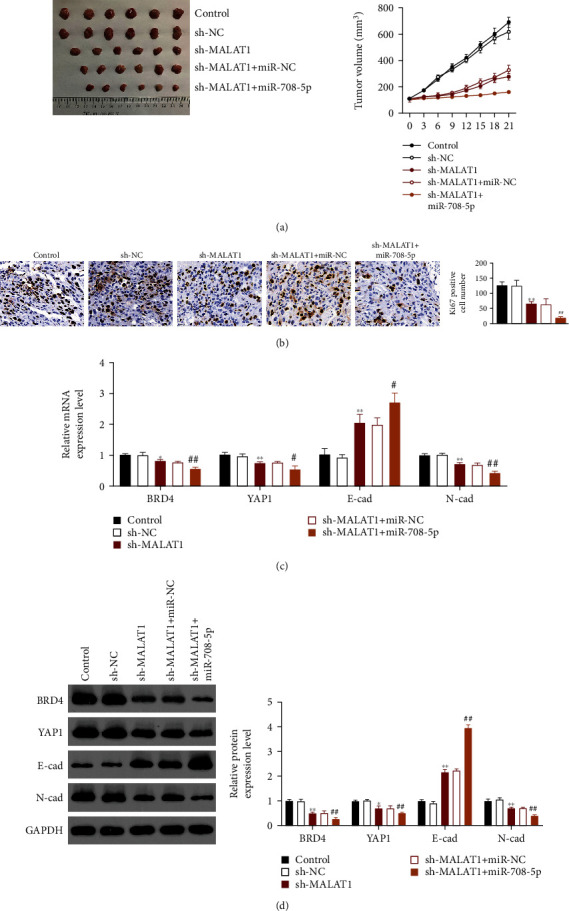
MALAT1/miR-708-5p regulated tumorigenicity *in vivo* by regulating BRD4 and YAP1-mediated EMT. (a) Tumor volume of different groups. (b) Immunohistochemistry (IHC) staining detected cell proliferation of different groups. (c) qRT-PCR showed the mRNA expression of BRD4, YAP1, E-cadherin, and N-cadherin in different groups. (d) Western blot assay revealed the protein expression of BRD4, YAP1, E-cadherin, and N-cadherin in different groups. ^∗^*P* < 0.05 and ^∗∗^*P* < 0.01 compared with the control group. ^#^*P* < 0.05 and ^##^*P* < 0.01 compared with the sh-MALAT1+miR-NC group.

To further confirm the role and regulatory mechanism of MALAT1 in laryngocarcinoma, we investigated the effects of MALAT suppression on tumorigenicity *in viv*o. As results, the volume of tumors was significantly decreased in the sh-MALAT1 group compared to that in the control group (*P* < 0.01, Figure 5(a)), which were further decreased in the sh-MALAT1+miR-708-5p ago group. At the same time, Ki67 expression was remarkably inhibited after suppression of MALAT1, which were further suppressed after MALAT1 suppression and miR-708-5p overexpression at the same time (Figure 5(b)). These data indicated that downregulation of MALAT1 remarkably suppressed tumorigenicity *in vivo*. Furthermore, downregulation of MALAT1 resulted in significant decreases in the BRD4, YAP1, and N-cadherin mRNA and protein expression and obvious increases in the E-cadherin mRNA and protein expression, which were further enhanced after MALAT1 suppression and miR-708-5p overexpression synchronously (*P* < 0.05, Figures 5(c) and 5(d)). These data indicated that MALAT1/miR-708-5p regulated tumorigenicity *in vivo* by regulating BRD4 and YAP1-mediated EMT.

## 4. Discussion

Abundant evidence has clarified that lncRNAs exhibit significant function in the biological progression of laryngocarcinoma by ceRNA network. For instance, upregulation of TUG1 facilitates tumor growth in laryngocarcinoma through targeting miR-145-5p/ROCK1 axis [20]. Downregulation of MIR22HG promotes malignant behaviors of laryngocarcinoma cells via regulating the miR-5000-3p/FBXW7 axis [21]. Therefore, our results were aimed at deciphering the ceRNA mechanism underlying MALAT1 in laryngocarcinoma.

In the present study, we found that MALAT1 suppression inhibited the malignant phenotypes of laryngocarcinoma cells, such as decreased proliferation, promoted apoptosis, and suppressed migration, and inhibited the CSC properties. Suppression of MALAT1 increased miR-708-5p expression and decreased the expression of BRD4. Moreover, there were target relationships between MALAT1 and miR-708-5p as well as between miR-708-5p and BRD4. Furthermore, both *in vitro* and *in vivo* experiments confirmed that MALAT1 regulated the malignant phenotypes of laryngocarcinoma cells and tumorigenicity by targeting miR-708-5p/BRD4 axis to regulate YAP1-mediated EMT.

LncRNAs have been shown to exert crucial effects on the tumorigenesis in multiple human cancers by sponging particular miRNA [22]. miR-708-5p is first identified in cancerous cervical samples, showing high sequence similarity to miR-28 [23]. miR-708-5p is found to be upregulated in pancreatic ductal adenocarcinoma (PDAC), and overexpression of miR-708-5p enhanced PDAC cell proliferation, invasion, and migration [24]. Dysregulation of miR-708-5p expression can enhance the protumorigenic phenotype in lung cancer cells by exacerbating oncogenic prostaglandin E2 production [25]. Feng et al. demonstrated that miR-708-5p impaired the EMT and metastasis of osteosarcoma [26]. However, the function of miR-708-5p in laryngocarcinoma has not been fully reported. Given the key role of miR-708-5p in various cancers, we speculate that miR-708-5p may also a key play in laryngocarcinoma. Moreover, our results found that MALAT1 could target miR-708-5p, and both *in vitro* and *in vivo* experiments confirmed that suppression of MALAT1 and miR-708-5p overexpression had synergistic inhibitory effects on the tumorigenicity of laryngocarcinoma cells. It can therefore be presumed that MALAT1 contributes to laryngocarcinoma by sponging miR-708-5p.

BRD4 is the most cancer-related BET family member and plays a pivotal role in certain types of cancer [27]. It is reported that BRD4 promote progression and metastasis of gastric cancer [28]. Lu et al. indicated that downregulation of BRD4 inhibits the malignancy of breast cancer cells [29]. Moreover, inhibition of BRD4 using sh-RNAs or the BET inhibitor JQ1 has been shown to result in dramatic antileukemic effects both *in vitro* and *in vivo* [30]. Notably, inhibition of BRD4 by the use of BET-inhibitors has been currently regarded as a promising strategy to target both hematological and solid malignancies [27]. Duan et al. also demonstrated that BRD4 was a promising anticancer drug target and suggested the clinical development and application of BRD4 inhibitors and degraders in anticancer treatment [31]. In this study, the results of dual-luciferase reporter assay showed that miR-708-5p could target BRD4, and suppression of MALAT1 and miR-708-5p overexpression had synergistic inhibitory effects on BRD4 expression. Therefore, we conclude that suppression of MALAT1 inhibits laryngocarcinoma by sponging miR-708-5p to target BRD4. Targeting BRD4 may also a promising strategy to treat laryngocarcinoma.

Strikingly, Song et al. showed that BRD4 is revealed as a critical regulator for YAP1 transcription and expression. JQ1 effectively inhibited YAP1 expression and transcription in human esophageal adenocarcinoma cells [17]. In our study, we also found that JQ1 could inhibit the mRNA and protein expression of YAP1. Moreover, YAP1 has exhibited oncoroles in a variety of cancer [32]. For instance, YAP1 has been proved to be involved in tumorigenic properties of tumor cells in colorectal cancer [33], prostate cancer [34], and gastric cancer [35]. In addition, overexpression of YAP1 can induce the activation of EMT [16], which is implicated in the migration and invasion in multiple cancers [36, 37]. TRPP2 is shown to promote metastasis in laryngeal squamous cell carcinoma by regulating EMT [38]. In our study, we also found that the decreased BRD4, YAP1, and N-cadherin expression and increased E-cadherin expression were observed after suppression of MALAT1, overexpression of miR-708-5p, or their combination. Therefore, we speculate that dysregulation of BRD4 could affect EMT via YAP1. YAP1-mediated EMT is a downstream mechanism of MALAT1/miR-708-5p/BRD4 axis to mediate the development of laryngocarcinoma.

In conclusion, our findings reveal that suppression of MALAT1 inhibits the development of laryngocarcinoma. MALAT1 may contribute to laryngocarcinoma development by sponging miR-708-5p to target BRD4. YAP1-mediated EMT is a downstream mechanism of MALAT1/miR-708-5p/BRD4 axis to mediate the development of laryngocarcinoma. Targeting MALAT1/miR-708-5p/BRD4 axis may provide a new insight for the development promising strategy to target laryngocarcinoma.

## Data Availability

The datasets used and analysed during the current study are available from the corresponding author on reasonable request.

## Additional Points

*Highlights*. (1) MALAT1 suppression inhibited the malignant phenotypes of laryngocarcinoma cells. (2) MALAT1 could sponge miR-708-5p to target BRD4 in laryngocarcinoma cells. (3) YAP1-mediated EMT is a key mechanism mediating laryngocarcinoma development. (4) MALAT1/miR-708-5p/BRD4 may be a promising strategy to target laryngocarcinoma

## Supplementary Materials

Supplementary Materials Supplementary Figure 1: the relative mRNA expression of MALAT1 was tested by qRT-PCR assay. ^∗∗^*P* < 0.01 compared with the sh-NC group.

## References

[1] Steuer C. E., El-Deiry M., Parks J. R., Higgins K. A., Saba N. F. (2017). An update on larynx cancer. *CA: a Cancer Journal for Clinicians*.

[2] Stewart B., Wild C. (2014). *World Cancer Report 2014*.

[3] Lampri E. S., Chondrogiannis G., Ioachim E. (2015). Biomarkers of head and neck cancer, tools or a gordian knot?. *International Journal of Clinical and Experimental Medicine*.

[4] Gu J., Han T., Sun L., Yan A.-H., Jiang X.-J. (2020). miR-552 promotes laryngocarcinoma cells proliferation and metastasis by targeting p53 pathway. *Cell Cycle*.

[5] Dinescu S., Ignat S., Lazar A. D., Constantin C., Neagu M., Costache M. (2019). Epitranscriptomic signatures in lncRNAs and their possible roles in cancer. *Genes*.

[6] Begolli R., Sideris N., Giakountis A. (2019). LncRNAs as chromatin regulators in cancer: from molecular function to clinical potential. *Cancers*.

[7] Denaro N., Merlano M. C., Nigro C. L. (2019). Long noncoding RNAs as regulators of cancer immunity. *Molecular Oncology*.

[8] Xu S., Guo J., Zhang W. (2019). lncRNA PCAT19 promotes the proliferation of laryngocarcinoma cells via modulation of the miR-182/PDK4 axis. *Journal of Cellular Biochemistry*.

[9] Liu T., Wang W., Xu Y. C., Li Z. W., Zhou J. (2019). Long noncoding RNA NEAT1 functions as an oncogene in human laryngocarcinoma by targeting miR-29a-3p. *European Review for Medical and Pharmacological Sciences*.

[10] He G., Yao W., Li L., Wu Y., Feng G., Chen L. (2020). LOXL1-AS1 contributes to the proliferation and migration of laryngocarcinoma cells through miR-589-5p/TRAF6 axis. *Cancer Cell International*.

[11] Chandra Gupta S., Nandan T. Y. (2017). Potential of long non-coding RNAs in cancer patients: from biomarkers to therapeutic targets. *International Journal of Cancer*.

[12] Bhan A., Soleimani M., Mandal S. S. (2017). Long noncoding RNA and cancer: a new paradigm. *Cancer Research*.

[13] Goyal B., Yadav S. R. M., Awasthee N., Gupta S., Kunnumakkara A. B., Gupta S. C. (2021). Diagnostic, prognostic, and therapeutic significance of long non-coding RNA MALAT1 in cancer. *Biochimica Et Biophysica Acta. Reviews on Cancer*.

[14] Nanni S., Aiello A., Salis C. (2021). Metabolic reprogramming by MALAT 1 depletion in prostate cancer. *Cancers*.

[15] Shibata M., Ham K., Hoque M. O. (2018). A time for YAP1: tumorigenesis, immunosuppression and targeted therapy. *International Journal of Cancer*.

[16] Yao P., Li Y., Shen W. (2018). ANKHD1 silencing suppresses the proliferation, migration and invasion of CRC cells by inhibiting YAP1-induced activation of EMT. *American Journal of Cancer Research*.

[17] Song S., Li Y., Xu Y. (2020). Targeting Hippo coactivator YAP1 through BET bromodomain inhibition in esophageal adenocarcinoma. *Molecular Oncology*.

[18] Sanchez-Mejias A., Tay Y. (2015). Competing endogenous RNA networks: tying the essential knots for cancer biology and therapeutics. *Journal of Hematology & Oncology*.

[19] Kartha R. V., Subbaya S. (2014). Competing endogenous RNAs (ceRNAs): new entrants to the intricacies of gene regulation. *Frontiers in Genetics*.

[20] Zhuang S., Liu F., Wu P. (2019). Retracted: upregulation of long noncoding RNA TUG1 contributes to the development of laryngocarcinoma by targeting miR-145-5p/ROCK1 axis. *Journal of Cellular Biochemistry*.

[21] Chen H., Ali M., Ruben A., Stelmakh D., Pak M. (2020). E2F6-mediated downregulation of MIR22HG facilitates the progression of laryngocarcinoma by targeting the miR-5000-3p/FBXW7 Axis. *Molecular and Cellular Biology*.

[22] Fang Y., Fullwood M. J. (2016). Roles, functions, and mechanisms of long non-coding RNAs in cancer. *Genomics, Proteomics & Bioinformatics*.

[23] Lui W. O., Pourmand N., Patterson B. K., Fire A. (2007). Patterns of known and novel small RNAs in human cervical cancer. *Cancer Research*.

[24] Huang S., Guo H., Cao Y., Xiong J. (2019). MiR-708-5p inhibits the progression of pancreatic ductal adenocarcinoma by targeting Sirt3. *Pathology, Research and Practice*.

[25] Monteleone N. J., Lutz C. S. (2020). miR-708-5p targets oncogenic prostaglandin E2 production to suppress a pro-tumorigenic phenotype in lung cancer cells. *Oncotarget*.

[26] Feng T., Zhu Z., Jin Y. (2020). The microRNA-708-5p/ZEB1/EMT axis mediates the metastatic potential of osteosarcoma. *Oncology Reports*.

[27] Donati B., Lorenzini E., Ciarrocchi A. (2018). BRD4 and cancer: going beyond transcriptional regulation. *Molecular Cancer*.

[28] Qin Z. Y., Wang T., Su S. (2019). BRD4 promotes gastric cancer progression and metastasis through acetylation-dependent stabilization of snail. *Cancer Research*.

[29] Lu L., Chen Z., Lin X. (2020). Inhibition of BRD4 suppresses the malignancy of breast cancer cells via regulation of Snail. *Cell Death and Differentiation*.

[30] Zuber J., Shi J., Wang E. (2011). RNAi screen identifies Brd4 as a therapeutic target in acute myeloid leukaemia. *Nature*.

[31] Duan Y., Guan Y., Qin W., Zhai X., Yu B., Liu H. (2018). Targeting Brd4 for cancer therapy: inhibitors and degraders. *Medchemcomm*.

[32] Seton-Rogers S. (2014). All eyes on YAP1. *Nature Reviews. Cancer*.

[33] Ma X., Zhang H., Xue X., Shah Y. M. (2017). Hypoxia-inducible factor 2*α* (HIF-2*α*) promotes colon cancer growth by potentiating Yes-associated protein 1 (YAP1) activity. *The Journal of Biological Chemistry*.

[34] Collak F. K., Demir U., Sagir F. (2020). YAP1 is involved in tumorigenic properties of prostate cancer cells. *Pathology Oncology Research*.

[35] Sun D., Li X., He Y. (2016). YAP1 enhances cell proliferation, migration, and invasion of gastric cancer in vitro and in vivo. *Oncotarget*.

[36] Yang J., Weinberg R. A. (2008). Epithelial-mesenchymal transition: at the crossroads of development and tumor metastasis. *Developmental Cell*.

[37] Thompson E. W., Newgreen D. F. (2005). Carcinoma invasion and metastasis: a role for epithelial-mesenchymal transition?. *Cancer Research*.

[38] Wu K., Shen B., Jiang F. (2016). TRPP2 enhances metastasis by regulating epithelial-mesenchymal transition in laryngeal squamous cell carcinoma. *Cellular Physiology and Biochemistry*.

