# Supplementary figures and images for: Long Noncoding RNA MALAT1 Promotes Laryngocarcinoma Development by Targeting miR-708-5p/BRD4 Axis to Regulate YAP1-Mediated Epithelial-Mesenchymal Transition
Source: Biomed Res Int. 2022 May 12;2022:8093949. doi: 10.1155/2022/8093949 (PMC9119785; doi:10.1155/2022/8093949)

# The relative mRNA expression of MALAT1

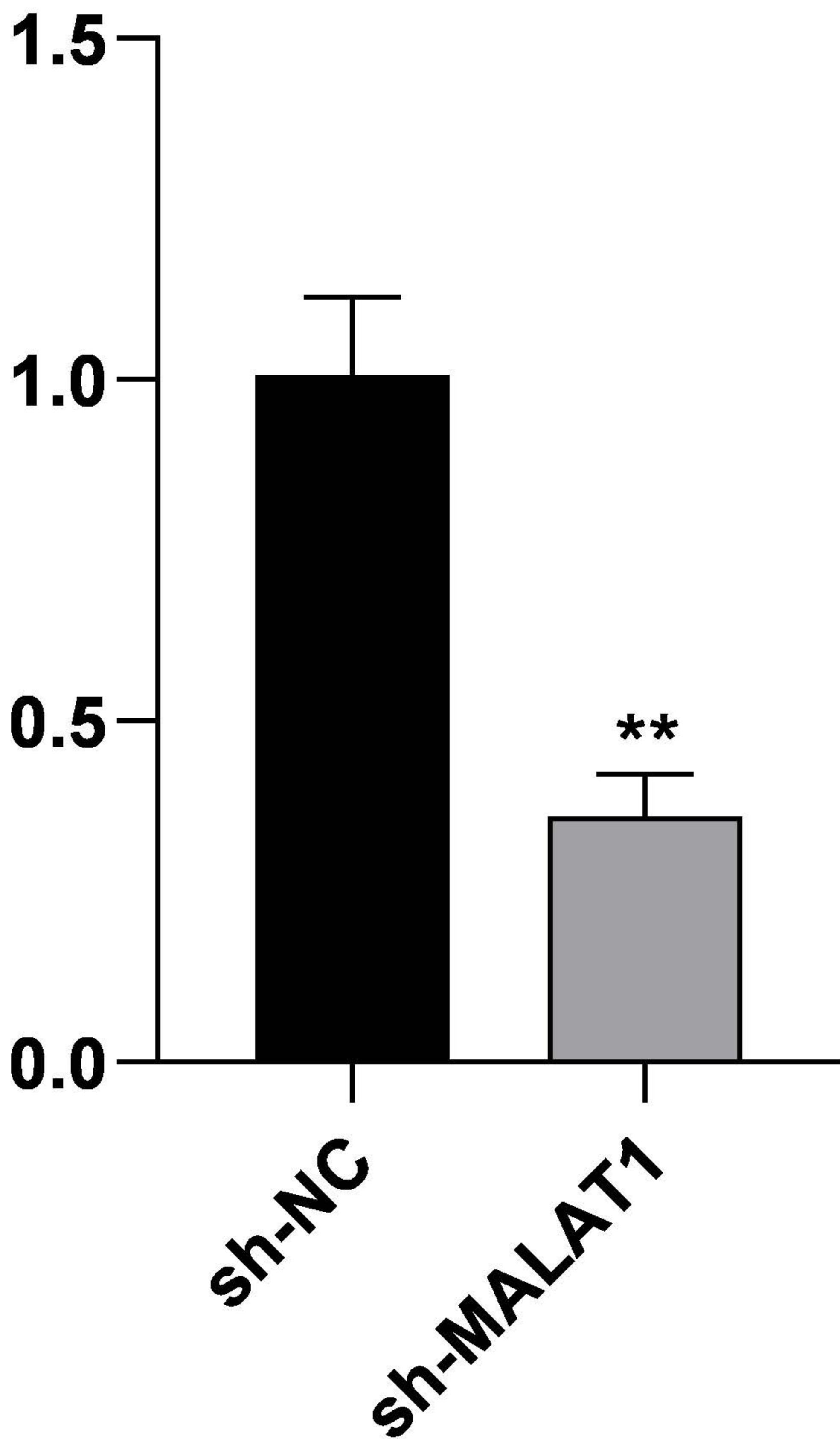

Supplement: Supplementary Materials — Supplementary Figure 1: the relative mRNA expression of MALAT1 was tested by qRT-PCR assay. ∗∗P < 0.01 compared with the sh-NC group. [file 8093949.f1.pdf]
